# Otoprotection to Implanted Cochlea Exposed to Noise Trauma With Dexamethasone Eluting Electrode

**DOI:** 10.3389/fncel.2019.00492

**Published:** 2019-11-22

**Authors:** Adrien A. Eshraghi, Amit Wolfovitz, Rasim Yilmazer, Carolyn Garnham, Ayca Baskadem Yilmazer, Esperanza Bas, Peter Ashman, Jonathan Roell, Jorge Bohorquez, Rahul Mittal, Roland Hessler, Daniel Sieber, Jeenu Mittal

**Affiliations:** ^1^Department of Otolaryngology, University of Miami Hearing Research Laboratory, Miller School of Medicine, Miami, FL, United States; ^2^Department of Neurological Surgery, Miller School of Medicine, Miami, FL, United States; ^3^Department of Biomedical Engineering, University of Miami, Miami, Coral Gables, FL, United States; ^4^MED-EL Hearing Implants, Innsbruck, Austria

**Keywords:** drug-eluting electrodes, otoprotection, noise trauma, hearing loss, local drug delivery, dexamethasone, cochlear implantation

## Abstract

Cochlear implantation (CI) is now widely used to provide auditory rehabilitation to individuals having severe to profound sensorineural hearing loss (SNHL). However, CI can lead to electrode insertion trauma (EIT) that can cause damage to sensory cells in the inner ear resulting in loss of residual hearing. Even with soft surgical techniques where there is minimal macroscopic damage, we can still observe the generation of molecular events that may initiate programmed cell death *via* various mechanisms such as oxidative stress, the release of pro-inflammatory cytokines, and activation of the caspase pathway. In addition, individuals with CI may be exposed to noise trauma (NT) due to occupation and leisure activities that may affect their hearing ability. Recently, there has been an increased interest in the auditory community to determine the efficacy of drug-eluting electrodes for the protection of residual hearing. The objective of this study is to determine the effect of NT on implanted cochlea as well as the otoprotective efficacy of dexamethasone eluting electrode to implanted cochlea exposed to NT in a guinea pig model of CI. Animals were divided into five groups: EIT with dexamethasone eluting electrode exposed to NT; EIT exposed to NT; NT only; EIT only and naïve animals (control group). The hearing thresholds were determined by auditory brainstem recordings (ABRs). The cochlea was harvested and analyzed for transcript levels of inflammation, apoptosis and fibrosis genes. We observed that threshold shifts were significantly higher in EIT, NT or EIT + NT groups compared to naive animals at all the tested frequencies. The dexamethasone eluting electrode led to a significant decrease in hearing threshold shifts in implanted animals exposed to NT. Proapoptotic tumor necrosis factor-α [TNF-α, TNF-α receptor 1a (TNFαR1a)] and pro-fibrotic transforming growth factor β1 (TGFβ) genes were more than two-fold up-regulated following EIT and EIT + NT compared to the control group. The use of dexamethasone releasing electrode significantly decreased the transcript levels of pro-apoptotic and pro-fibrotic genes. The dexamethasone releasing electrode has shown promising results for hearing protection in implanted animals exposed to NT. The results of this study suggest that dexamethasone releasing electrode holds great potential in developing effective treatment modalities for NT in the implanted cochlea.

## Introduction

Cochlear implantation (CI) is long known as the modality of choice for hearing rehabilitation in individuals with severe to profound sensorineural hearing loss (SNHL; Eshraghi et al., 2012; Lenarz et al., 2013; Macherey and Carlyon, 2014; Akçakaya et al., 2019). In recent years, CI indications have expanded to include candidates with substantial residual hearing and consequently, measures to preserve this residual hearing have become an important area of interest (Raveh et al., 2015; Eshraghi et al., 2017). The strategies for hearing preservation focus both on electrode design (softer, less traumatic and possibly drug-eluting) and on new surgical techniques that will enable minimal traumatic implantation (Eshraghi et al., 2011; Miranda et al., 2014; Khater and El-Anwar, 2017). The mechanisms involved in the loss of residual hearing following CI have been studied and demonstrate early and delayed loss of this hearing (Eshraghi et al., 2005; Dedhia et al., 2016; Quesnel et al., 2016). Early loss of residual hearing starts soon after the introduction of the electrode into cochlea that may lead to direct structural damage and consequent necrosis of cochlear sensory cells (Jia et al., 2013). This process is followed by the initiation of extrinsic and intrinsic molecular pathways of cochlear damage (Eshraghi and Van de Water, 2006). These pathways are triggered by oxidative stress and the expression of pro-inflammatory cytokines (Dinh et al., 2015). These cytokines, and oxidative damage, in turn, lead to activation of pro-apoptotic pathways, mitogen-activated protein kinases/c-Jun-N-terminal kinases (MAPK/JNK), that lead to programmed cell death in the affected cells within the first 24 h following electrode insertion trauma (EIT; Eshraghi et al., 2010, 2015). These pathways may continue with expression of pro-fibrogenic cytokines that produce fibrogenesis and angiogenesis beginning around 96 h post-implantation. The activation of these inflammatory and apoptotic pathways may further be accelerated by exposure of implanted individuals to noise trauma (NT). This NT may include both acoustic NT and electric overstimulation. There is a growing number of people with CI for whom the effect of NT is unclear, compared to the non-implanted population. NIHL has been reported as the etiology of deafness in implanted individuals, with a prevalence ranging from 2% (CI) to 20% (CI with electroacoustic stimulation; Simpson et al., 1993; Lazard et al., 2012). Currently, we can only speculate on the extent to which the SNHL in these implanted individuals can be attributed to noise exposure or due to a combination of other underlying predisposing factors. For example, implanted individuals may be exposed to high levels of noise due to occupation and leisure activities such as attending musical concerts. However, the effect of this NT on the hearing ability of implanted individuals is still not known. Therefore, the objective of the present study is to assess the effect of NT on the hearing in an implanted cochlea with and without dexamethasone eluting electrodes.

## Materials and Methods

### Experimental Groups

**Figure 1:**
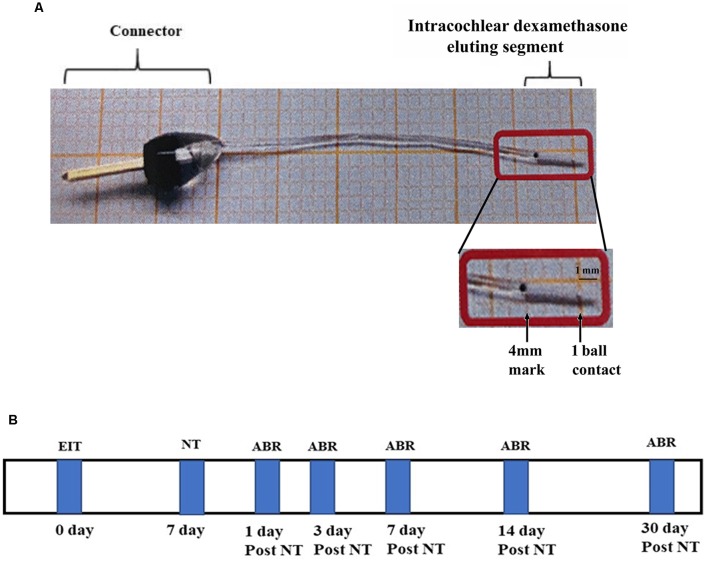
Drug-eluting electrode and timeline. **(A)** Dexamethasone eluting electrode showing a connector and intracochlear segment with one electrical ball contact at the distal end. **(B)** Experimental timeline.

Both male and female pigmented guinea pigs (Cady Hill Farms, MA) weighing approximately 350 g were used in this study. Animals were divided into five groups: EIT with dexamethasone eluting electrode (10%) exposed to NT (*n* = 13); EIT exposed to NT (*n* = 13); NT only (*n* = 13); EIT only (*n* = 13) and naïve animals separate for EIT or NT group and EIT/NT or EIT/NT/Dex group (control group; *n* = 13 each separately for these two different groups). The separate group of control animals was used for EIT or NT group and EIT/NT or EIT/NT/Dex group to avoid unnecessary exposure of animals to auditory brainstem recording (ABR) recordings. In the EIT or NT group, ABRs were done on days 1, 3, 7, 14 and 30 after implantation or exposing to high noise whereas in EIT/NT or EIT/NT/Dex group, the animals were first implanted, subjected to NT 7 days post-implantation and then ABRs were performed. A separate cohort of animals was used for gene expression (RT-PCR) studies (*N* = 6 for each above mentioned five groups). The production and physical characteristics of dexamethasone eluting electrodes have been described in detail in our previous studies (Bas et al., 2016). The electrode was made up of a sterile silicone rod with one recording contact at 1 mm from the tip (Figure 1A). It was designed for the guinea pig basal turn with a diameter of 0.3 mm at the tip, increasing gradually in size to be 0.5 mm in diameter at a distance of 4 mm from the tip. After implantation, dexamethasone from the drug-eluting electrode is released into the surrounding perilymph in the cochlea. The dexamethasone release rate from 10% eluting electrode has been demonstrated to be higher during the initial period of 5 days (166 ng/day) that goes down to 49 ng/day by 91 day determined by high performance liquid chromatography-mass spectrometry (HPLC-MS) in an *in vitro* study (Wilk et al., 2016). Another *in vitro* study also confirmed the comparable levels and similar patterns of dexamethasone release from drug-eluting electrode using a similar approach (Bas et al., 2016). For NT, awake animals were exposed to the sound level of 120 dB, 6–10 kHz centered at 8 kHz for 2 h. A number of studies have exposed awake animals to high NT without any undue pain and distress to the animals (Housley et al., 2013; Yang et al., 2015; Tuerdi et al., 2017; Vlajkovic et al., 2017; Chen et al., 2019). For noise exposure, animals were kept in individual cages and placed in an electrically shielded, double-walled sound-attenuating chamber. The sound was generated by a waveform generator, amplified by an audio amplifier (Pioneer Electronics, Long Beach, CA, USA), and presented in an open field through speakers (Pioneer Electronics, Long Beach, CA, USA) placed 10 cm in front of the animal’s cage. The noise level was determined using sound meter (Extech, Waltham, MA, USA) that measures sound from 35 to 130 dB. Animals were subjected to NT for 7 days post-CI surgery. Hearing thresholds of animals were determined at various time-periods before and after the surgery or NT. The experimental timeline of this study has been shown in Figure 1B.

All animal procedures used in this study were approved by the University of Miami Institutional Animal Care and Use Committee and followed NIH guidelines for the use and care of animals in biomedical research.

### Surgical Procedures

The guinea pigs were anesthetized with intraperitoneal ketamine (40 mg/kg) and xylazine (5 mg/kg). Local anesthesia was administered with a 1% lidocaine subcutaneous injection. The operated ear was randomly assigned for implantation. Using sterile techniques and a binocular operating microscope, a retro-auricular incision was made to expose the bulla of the experimental ear. The bulla was opened with a scalpel, the round window membrane (RWM) and promontory were identified, and a cochleostomy was performed in the basal turn of the cochlea using a diamond drill of 0.8 mm. An electrode (dexamethasone eluting or non-eluting) was slowly inserted *via* the cochleostomy for a length of 4 mm into the scala tympani as described in our previous studies (Eshraghi et al., 2013, 2015; Bas et al., 2016). The electrode was then secured in place with a fascia graft at the cochleostomy site. Once the stability of the électrode array was established, the defect in the temporal bone bulla was closed with carboxylate dental cement. A burr hole was made in the skull at 1 mm anterior to the lambda suture and a stainless steel screw was implanted superficial to the dura (“epidural”) to record auditory brainstem responses. The post-auricular incision was closed using several interrupted sutures. Intraperitoneal buprenorphine (0.05 mg/kg) was given for analgesia at the time of surgery and twice a day for two more days post-surgery.

### Auditory Brainstem Recording (ABR)

The hearing of both ears of all guinea pigs was measured by ABR responses to pure-tone stimuli (1, 4, 8, and 16 kHz). The ABR frequencies were selected based on previous studies (Eshraghi et al., 2013; Bas et al., 2016). The ABRs were obtained using recording electrodes placed in the superior postauricular area (−) and in the vertex (+) of the guinea pig’s scalp. The ground electrode was inserted in a deep muscle of the left leg. Intelligent Hearing Systems (IHS Smart EP, Miami, FL, USA) hardware and software were used to record ABR responses. Stimuli were delivered at a rate of 29 Hz to the cochlea tested by using an Etymotic Research ER2 insert earphone (Etymotic Research, Elk Grove Village, IL, USA) with a custom-made silicone ear tip that fits the guinea pig’s ear canal diameter. The responses were amplified using an Opti-Amp bio amplifier from IHS that was connected to the Smart EP system. The intensity of stimulation was decreased by 10-dB decrements until no ABR response was identifiable by software developed by IHS to establish threshold values. These threshold values were confirmed by visual inspection of the responses by two of the investigators blinded to the study groups.

### Quantitative RT-PCR Analyses

Total RNA was extracted from whole cochlear tissues with TRIzol reagent (Invitrogen, Carlsbad, CA, USA) following the manufacturer’s protocol. RNA purity and concentration were determined by the absorbance at 260 and 280 nm using NanoDrop ND-1000 (Thermo Fisher Scientific, Waltham, MA, USA; Bas et al., 2019). cDNA was synthesized using an iScript kit (Bio-Rad, Hercules, CA, USA). Quantitative real-time PCR was performed in duplicate by using iQ SYBR Green Supermix (Bio-Rad, Hercules, CA, USA) on an iCycler Real-Time CFX96 detection system (Bio-Rad, Hercules, CA, USA). The mRNA level was normalized by using the housekeeping gene GAPDH. The primers were designed based on the cDNA sequences obtained from Ensemble Genome Browser^1^ and NCBI nucleotide database^2^ as described in previous studies (Bas et al., 2012). Melting curves were also performed to ensure primer specificity and to evaluate for any contamination. Relative changes in mRNA levels of genes were assessed using the 2(−△△ CT) method (Livak and Schmittgen, 2001) and normalized to the house-keeping gene GAPDH. They were then normalized to the expression levels obtained from the control. Six whole cochlear tissue explants per group were used for each time point and three independent replicates were done. The average fold change compared to control group was calculated.

### Statistical Analysis

Two-way ANOVA with Bonferroni *post hoc* testing was used for ABR threshold comparisons between groups at different time points. For gene expression studies, student’s *t*-test was used. *P* < 0.05 was considered statistically significant.

## Results

### Effect of Dexamethasone Eluting Electrodes on Hearing Thresholds in a Guinea Pig Model

**Figure 2:**
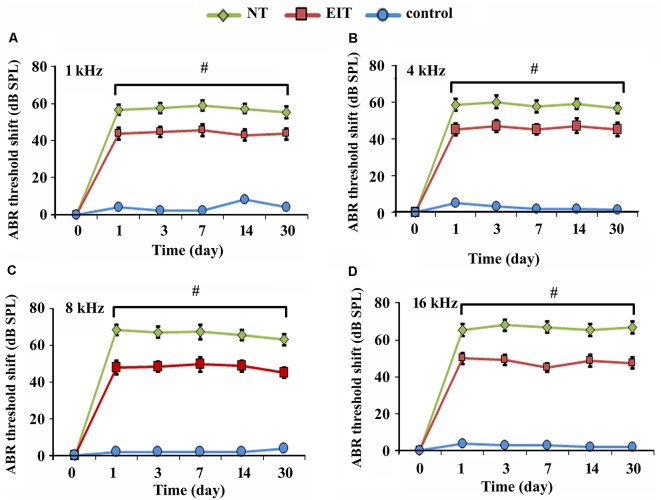
Auditory brainstem recording (ABR) threshold shifts. Hearing threshold shifts in control, electrode insertion trauma (EIT) group, and animals exposed to noise trauma (NT) at 1 kHz **(A)**, 4 kHz **(B)**, 8 kHz **(C)** and 16 kHz **(D)** at different time-periods. ABR threshold shifts were significantly higher in NT groups compared to implanted animals. ^#^*P* < 0.05 compared to the EIT group. Data represent mean ± standard deviation.

To determine the effect of EIT and NT on hearing, guinea pigs were subjected to ABRs. Unimplanted naïve animals served as the control group. In agreement with our previous studies, hearing threshold shifts were significantly higher in implanted animals compared to the control group at 1, 4, 8 and 16 kHz at all post-implanted time periods (*P* < 0.001; Figures 2A–D). The threshold shifts were significantly higher in the noise-exposed unimplanted animals compared to the group with an implanted cochlea (EIT) at all tested frequencies (*P* < 0.05).

**Figure 3:**
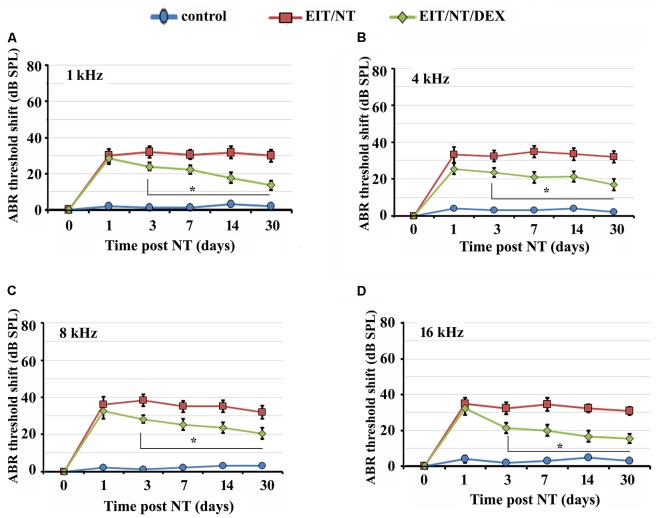
Dexamethasone eluting electrodes provides otoprotection. Guinea pigs were implanted with dexamethasone eluting or non-eluting electrode and subjected to NT. ABR thresholds were significantly lower in animals implanted with dexamethasone eluting electrodes compared to guinea pigs receiving non-eluting electrodes at 1 kHz **(A)**, 4 kHz **(B)**, 8 kHz **(C)** and 16 kHz **(D)** at all time-periods. Time period zero indicates baseline ABRs before EIT and NT. *P < 0.01 compared to EIT/NT group. Data represent mean ± standard deviation.

Next, we determined the hearing thresholds of animals implanted with non-eluting or dexamethasone eluting electrodes. We observed higher threshold shifts in animals that were exposed to NT with non-eluting electrodes at all tested frequencies compared to the control group (Figures 3A–D; *P* < 0.001). Threshold shifts in the animals implanted with dexamethasone eluting electrodes and subjected to NT (7 days after post-CI surgery) were consistently smaller compared to the threshold shift that was observed in guinea pigs with the non-eluting electrodes exposed to NT starting from day 3 up to day 30 (Figures 3A–D; *P* < 0.01). These results suggest that dexamethasone provides otoprotection against NT and EIT.

### Changes in Transcript Levels of Cytokine and Receptors in Cochleae of Guinea Pigs Exposed to NT

**Figure 4:**
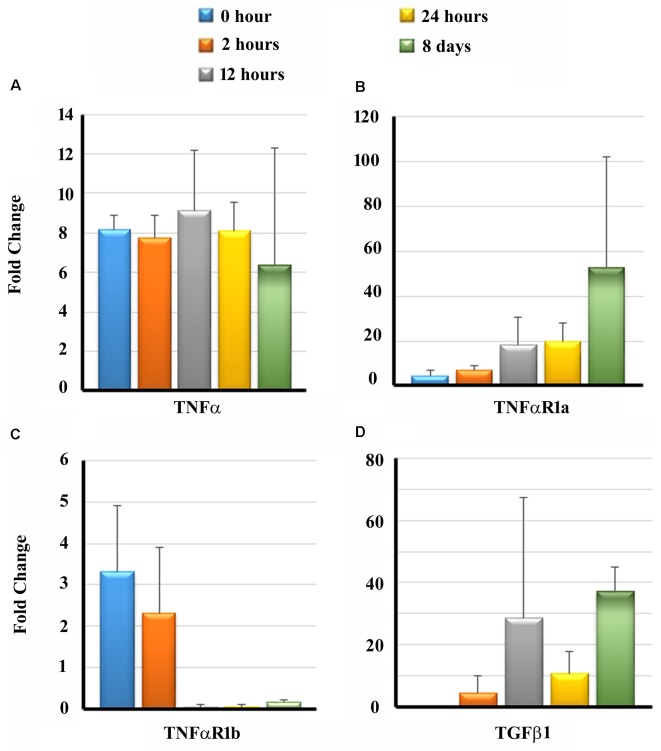
Transcripts levels of pro-inflammatory and pro-fibrogenic genes in the cochleae. Guinea pigs were subjected to NT and cochleae were harvested at different post-NT time periods. The expression of tumor necrosis factor-α (TNF-α; **A**), TNF-α receptor 1a (TNFαR1a; **B**), TNFαR1b **(C)** and transforming growth factor β1 (TGF β1; **D**) was determined by real-time PCR. Time zero represents cochleae harvested immediately after NT. Data are expressed as fold change compared to the control group and represents mean ± standard deviation.

Previous studies have demonstrated that cytokines, namely tumor necrosis factor-α (TNF-α) and transforming growth factor β1 (TGF β1), play a significant role in the initiation of apoptotic pathways and fibrosis leading to sensory cell damage following cochlear insults (Dinh et al., 2015; Bas et al., 2019). The binding of TNF-α to its receptors, TNF-α receptor 1a (TNFαR1a) and TNF-α receptor 1b (TNFαR1b), can initiate downstream host signaling cascade leading to apoptosis of auditory hair cells (Morrill and He, 2017). Therefore we determined the transcript levels of TNFα and TGF β1 as well as TNFαR1a and TNFαR1b in the cochlear tissues of guinea pigs exposed to NT using real-time PCR at different time periods post-NT. There was a significant upregulation in transcript levels of TNFα at 2 and 12 h post-NT, followed by a slight decrease in levels at 24 h and 8 days post-NT (Figure 4A). Increased transcript levels of TNFαR1a were observable with an increase in the post-NT time period from 2 h to 8 days (Figure 4B). TNFαR1b was initially significantly up-regulated in the first 2 h post NT followed by decreased levels in the later time intervals (Figure 4C). TGF β1 showed the highest transcript levels at 8-day post-NT. These results suggest that NT induces significant upregulation of inflammatory and fibrotic gene expression in the cochlea exposed to NT (Figure 4D).

### Expression of Cytokine and Receptors in Cochleae From Implanted Animals

**Figure 5:**
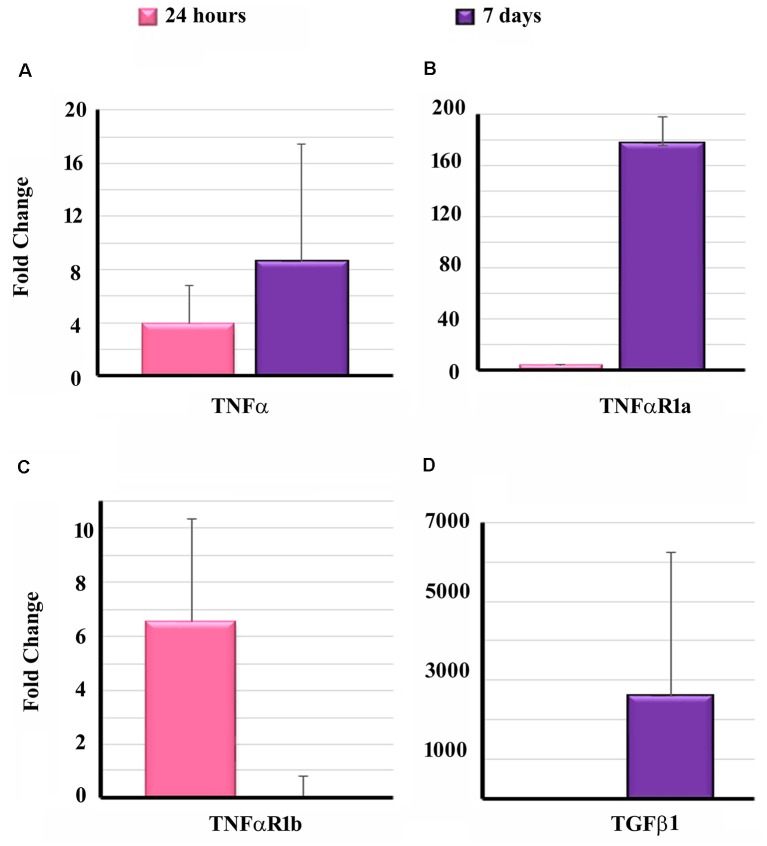
Expression of pro-inflammatory and pro-fibrogenic genes in the cochleae of implanted animals. Cochleae from guinea pigs implanted with non-drug eluting electrodes were harvested at the different post-EIT time period and subjected to real-time PCR to determine the levels of TNF-α **(A)**, TNFαR1a **(B)**, TNFαR1b **(C)** and TGF β1 **(D)**. Data are expressed as fold change compared to the control group and represents mean ± standard deviation.

To determine the effect of EIT on cytokine and receptors, the transcript levels of TNF-α, TNFαR1a, TNFαR1b and TGF β1 were determined in the cochlear tissues of implanted animals at 24 h and 7-day post-EIT. High transcript levels of TNF-α were observable at 24 h post-EIT showing significantly elevated expression at 7-day post-EIT (Figure 5A). A similar pattern of expression of TNFαR1a was observed showing high production at 7-day post-EIT (Figure 5B). On the other hand, elevated levels of TNFαR1b were observed only at 24 h post-EIT, followed by decreased expression at 7-day post-EIT (Figure 5C). High levels of TGF β1 were observable at 7-day post-EIT that are consistent with its role in fibrosis and scar formation following electrode insertion (Figure 5D).

### DEX Eluting Electrode Significantly Decreases the Transcript Levels of Cytokine and Receptors

**Figure 6:**
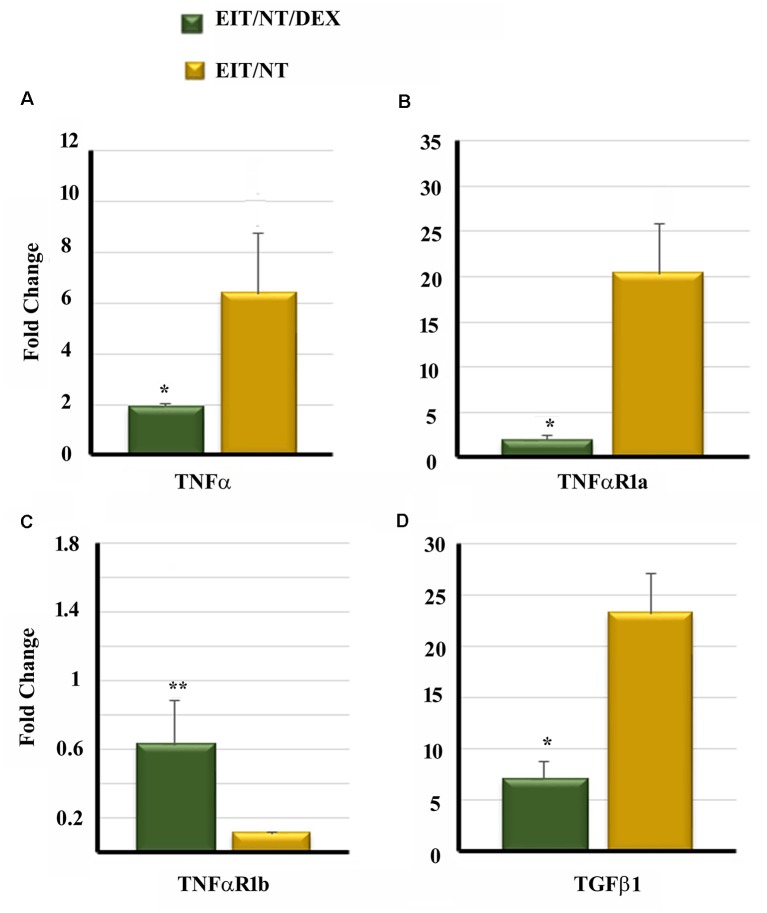
Dexamethasone significantly decreases the expression of pro-inflammatory and pro-fibrogenic genes in the cochleae. Guinea pigs were implanted with dexamethasone or non-drug-eluting electrodes and subjected to NT. Cochleae were harvested at different time periods. The expression of TNF-α **(A)**, TNFαR1a **(B)**, TNFαR1b **(C)** and TGF β1 **(D)** was determined by real-time PCR. *P < 0.01 or **P > 0.05 compared to EIT/NT group. Data are expressed as fold change compared to control group and represents the mean ± standard deviation.

We compared the transcript levels of TNF-α, TNFαR1a, TNFαR1b and TGF β1 in the cochlear tissues of animals implanted with dexamethasone eluting and non-eluting electrodes exposed to NT. The cochlear tissues harvested from animals implanted with non-eluting electrodes and exposed to NT showed high transcript levels of TNF-α, TNFαR1a and TGF β1 (Figures 6A,B,D). However, a significant downregulation of TNF-α, TNFαR1a, and TGF β1 was observed in the cochlear tissues of guinea pigs implanted with dexamethasone eluting electrodes and subjected to NT (Figures 6A,B,D; *P* < 0.001). An insignificant increase in elevation of TNFαR1b was observed in the inner ear tissues from animals implanted with dexamethasone eluting electrode (*P* > 0.05; Figure 6C).

## Discussion

Macroscopic damage to the cochlea occurring right after implantation results in the activation of intrinsic and extrinsic molecular pathways in the cochlea leading to sensory cell damage (Eshraghi and Van de Water, 2006; Dinh et al., 2015). By 24 h, oxidative stress induced by mechanical trauma, pro-inflammatory cytokines (mainly TNFα), and enzymes are released (Eshraghi et al., 2015). The caspase pathway is then activated leading to a pro-apoptotic signal cascade and programmed cell death. The expression of pro-apoptotic molecules (such as c-Jun, p-Jun, and caspase 3) was observed in immunohistochemistry studies to be expressed in hair cells and supporting cells as early as 6 h post-EIT (Eshraghi et al., 2015). By 96 h, pro-inflammatory cytokines and enzymes return to baseline level and fibrogenesis is initiated by growth factors such as TGF β1. The activation of inflammatory and apoptotic pathways may further be exacerbated following exposure to acoustic NT or electric overstimulation. Since there is an ever-growing population of implanted individuals exposed to the unchecked noise levels of daily living, it is still unknown what would be the influence of these unchecked noise levels on those individuals with impaired inner ear anatomy and cochlear sound wave conduction. This influence of NT on implanted ears has not been assessed to the best of our knowledge.

Recently, there has been an increased interest in the auditory community for the delivery of otoprotective agents into the cochlea through drug-eluting electrodes. Mostly silicone polymer is used for the delivery of otoprotective agents that are embedded into the electrode array (Farahmand-Ghavi et al., 2010). The utility of a drug-eluting electrode was initially demonstrated by an *in vitro* study (Paasche et al., 2003). Using a modified electrode attached to a pump, it was shown that it is possible to deliver the drugs in temporal bones, which was used as a surrogate model of the human cochlea. Another study showed improvement in hearing thresholds as evident by ABR and distortion product otoacoustic emission (DPOAE) values in guinea pigs implanted with 2% dexamethasone eluting rods compared to animals having non-eluting control rods (Liu et al., 2015). The cochleae of animals that received dexamethasone eluting rods also showed less fibrosis and lower levels of TNFα compared to the control group. Pharmacokinetic studies demonstrated peak dexamethasone concentration at 30 min postoperatively. It was observed that dexamethasone eluting rods can deliver the drug up to 1-week post-implantation in *in vivo* studies. In agreement with these findings, another investigation using a guinea pig model showed that dexamethasone eluting electrode provided otoprotection to the cochlea against elevation in hearing thresholds, loss of hair cells, damage to neural elements, increases in impedance and fibrosis that result from EIT-initiated damage in a dose-dependent manner up to 90 days (Bas et al., 2016). It has also been shown that dexamethasone eluting electrode (1 and 10%) promotes short-term preservation of residual hearing 4–6 weeks post-implantation in a gerbil model (Douchement et al., 2015). However, in this study, the long-term preservation of residual hearing (after 1-year post-EIT) was observed only at higher frequencies (16,000 Hz) and not at lower frequencies (500 and 1,000 Hz). All these studies demonstrate the efficacy of dexamethasone eluting electrode in providing otoprotection and promoting the preservation of residual hearing following EIT. In our study, we observed that the dexamethasone eluting electrode not only provides otoprotection against EIT but also for NT in our guinea pig model.

It is also interesting to note that in the present study, we observed that ABR thresholds were lower in implanted animals subjected to NT compared to implanted animals or those exposed to NT alone Although the precise reasons for this effect are still not known, it is possible that implanted animals actually receive less sound due to the electrode in the cochlea compared to non-implanted animals. In addition, there is a release of growth factors as well as heat shock proteins (HSP) in the inner ear following CI (Warnecke et al., 2019). Higher insulin-like growth factor binding protein 1 (IGFBP-1) levels have been observed in the perilymph of implanted individuals (Warnecke et al., 2019). IGFBP1 regulates the action of IGF-1 that plays a crucial role in the embryonic and postnatal development of the cochlea (Camarero et al., 2001; Digicaylioglu et al., 2004; Bach, 2015). Studies have shown that IGF-1 can act as a potent otoprotective agent and provide protection against auditory hair cell damage (Hayashi et al., 2013, 2014; Yamamoto et al., 2014; Yamahara et al., 2015, 2019). The local administration of IGF-1 in a hydrogel provides protection against acoustic NT in both guinea pig and rat animal models (Iwai et al., 2006; Lee et al., 2007). The elevated levels of HSPs such as HSP70 have also been observed in the perilymph of implanted individuals (Schmitt et al., 2018). Geldanamycin-induced HSP70 has been demonstrated to provide otoprotection against gentamicin-mediated ototoxicity (Yu et al., 2009). This pre-availability of these growth factors and HSPs may account for the otoprotection observed in implanted animals exposed to NT. Further studies are warranted to understand the precise molecular mechanisms that provide otoprotection to implanted animals exposed to NT.

Cochlear insults such as EIT and NT induce the generation of proinflammatory cytokines such as TNFα (Fujioka et al., 2006). The binding of TNFα to its receptor, TNFαR1a, and TNFαR1b initiates downstream signaling cascades that can play a crucial role in sensory cell damage in the cochlea (Dinh et al., 2015). In this study, we observed that NT induced a significant up-regulation of TNFα and its receptor, TNFαR1a, within the first 24 h. TNFα levels remained relatively constant initially but gradually decreased 8 days post-implantation. On the other hand, TNFαR1a continued to be exponentially up-regulated even 8 days post-implantation, thus promoting pro-apoptotic pathways even at that time. TNFαR1b was significantly up-regulated within the first 2 h. It was then significantly down-regulated within 12 h post-NT. TNFα receptors are known to promote nuclear entry of the transcription factor NF-κB that can induce transcription of pro-inflammatory genes as well as activation of the apoptosis pathway (Aggarwal, 2003; Hayden and Ghosh, 2014). TGF β1, a strong mediator of fibrogenesis, inflammatory, and immune response in the cochlea (Bas et al., 2019), was significantly up-regulated within 2 h post-NT leading to a decrease in the pro-apoptotic pathways on the one hand but an increase in fibrosis on the other. EIT led to a significant up-regulation of inflammatory and fibrotic gene expression (TNFα, TNFαR1a, and TGF β1) in 24 h and even more significantly after 7 days. The inflammatory and fibrotic gene expression was significantly decreased with the dexamethasone eluting electrode. Our results are in agreement with a previous study that showed a significant reduction in recruitment of inflammatory cells such as fibrocyte, lymphocyte macrophage, and giant cells in the cochlea of animals implanted with a dexamethasone-loaded silicone elastomer shaped like a CI electrode when compared to animals receiving non-eluting electrode (Farhadi et al., 2013).

In summary, the present study demonstrated that both the ABR threshold shift and the expression of pro-apoptotic and pro-fibrogenic genes were significantly decreased in dexamethasone implanted animals exposed to NT compared to animals implanted with non-drug eluting electrodes or subjected to either NT or EIT alone. One of the limitations of the present study is that the NT used in this investigation is artificial and NT in CI patients may be of a different origin, leading to different pathological changes in the ear, which needs to be deciphered in future studies. However, our study is very close to the situations where implanted individuals are exposed to acoustic trauma such as while attending musical concerts. Another limitation of our study that we tested the hearing thresholds in our guinea pig model only at lower frequencies. The higher frequencies can provide information on direct trauma impact and therapeutic results of dexamethasone releasing electrode. This is relevant in view of the electrode position and dexamethasone release in the basal region of the cochlea. Further studies will help in determining the effect of NT on hearing thresholds at higher frequencies in the implanted cochlea. Despite these limitations, our study and published literature strongly suggest the potential of dexamethasone eluting electrodes in providing otoprotection and preservation of residual hearing post-CI. However, dexamethasone downregulates immune responses in the cochlea, which may increase predisposition to infections. Due to the proximity of the ear to the brain and the presence of cochlear as well as vestibular aqueduct, cochlear infections are a potential risk factor for developing meningitis. Therefore, long-term safety studies are warranted to determine the optimum doses of dexamethasone. In addition, there is a need to discover novel otoprotective agents that can be delivered through drug-eluting electrodes which can promote the preservation of residual hearing post-CI. Drug-eluting electrodes hold great translational potential for the delivery of therapeutics to the inner ear. Due to the complex anatomy of the inner ear, cochlear drug delivery is associated with significant challenges. It is expected that drug-eluting electrodes will revolutionize the field of drug delivery into the inner ear leading to the efficient administration of therapeutics into the cochlea. Based on the results of this study, it can be concluded that dexamethasone eluting rods can protect the cochlea from EIT and NT alone and also from a combination of EIT and NT in a guinea pig model. Further studies using a larger cohort of animals will help in confirming the results of the present study and will open up avenues for potential translation of drug-eluting electrodes from bench to bedside.

## Ethics Statement

All animal procedures used in this study were approved by the University of Miami Institutional Animal Care and Use Committee and followed NIH guidelines for the use and care of animals in biomedical research.

## Author Contributions

All authors listed have made a substantial, direct and intellectual contribution to the work, and approved it for publication.

## Conflict of Interest

AE is a consultant and received funding from MED-EL Corporation. CG, RH, and DS are employees of MED-EL Corporation. The remaining authors declare that the research was conducted in the absence of any commercial or financial relationships that could be construed as a potential conflict of interest. The reviewer VS declared a past co-authorship with one of the authors RH to the handling Editor.

## Footnotes

^1^http://www.ensembl.org

^2^http://www.ncbi.nlm.nih.gov/nuccore
